# Supplementary figures and images for: Analysis of the Interactome of the Toxoplasma gondii Tgj1 HSP40 Chaperone
Source: Proteomes. 2023 Mar 1;11(1):9. doi: 10.3390/proteomes11010009 (PMC10056330; doi:10.3390/proteomes11010009)

## Slide 1
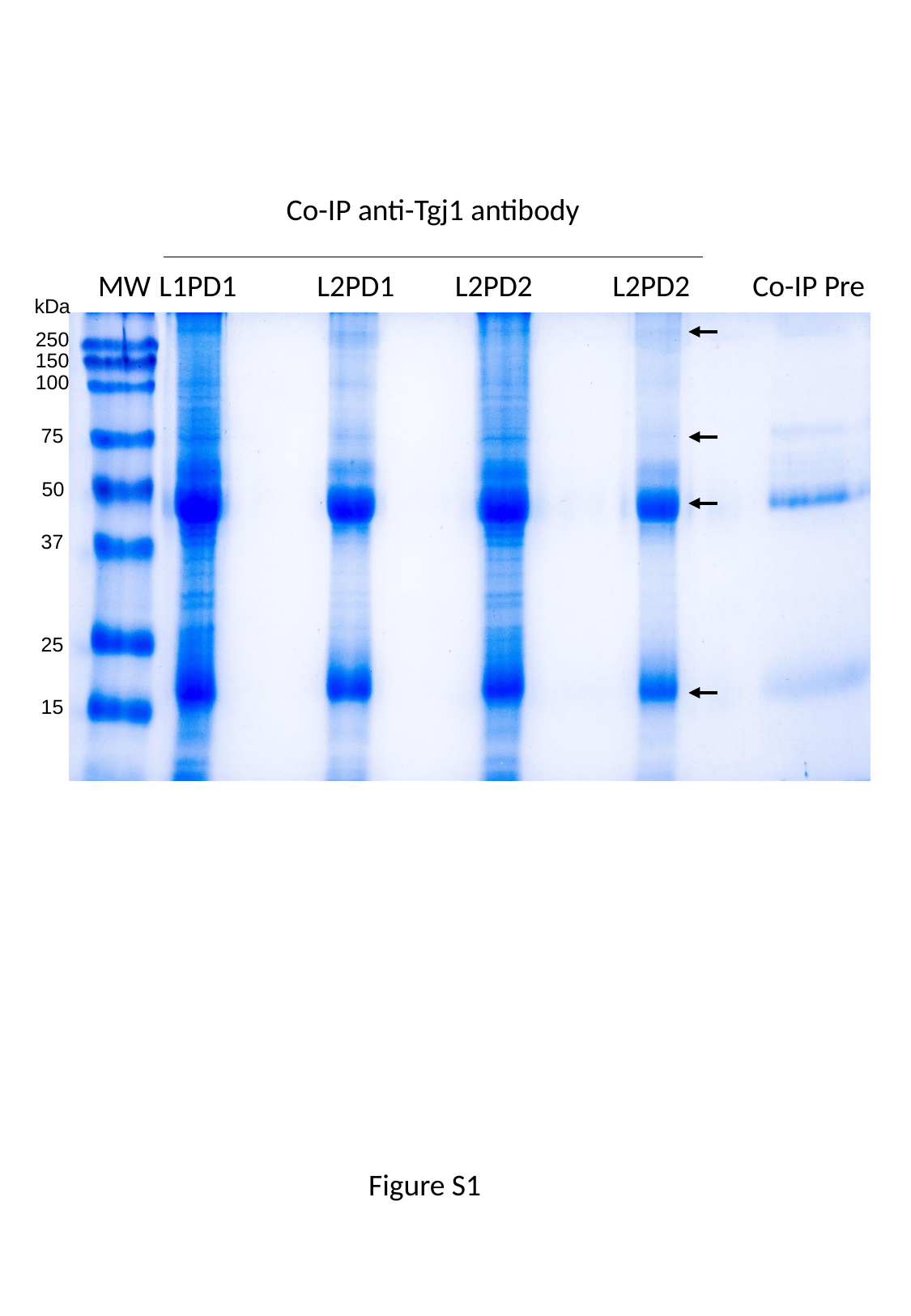

Co-IP anti-Tgj1 antibody
MW
L1PD1
L2PD1
L2PD2
L2PD2
Co-IP Pre
50
kDa
250
150
100
75
37
25
15
Figure S1

## Slide 2
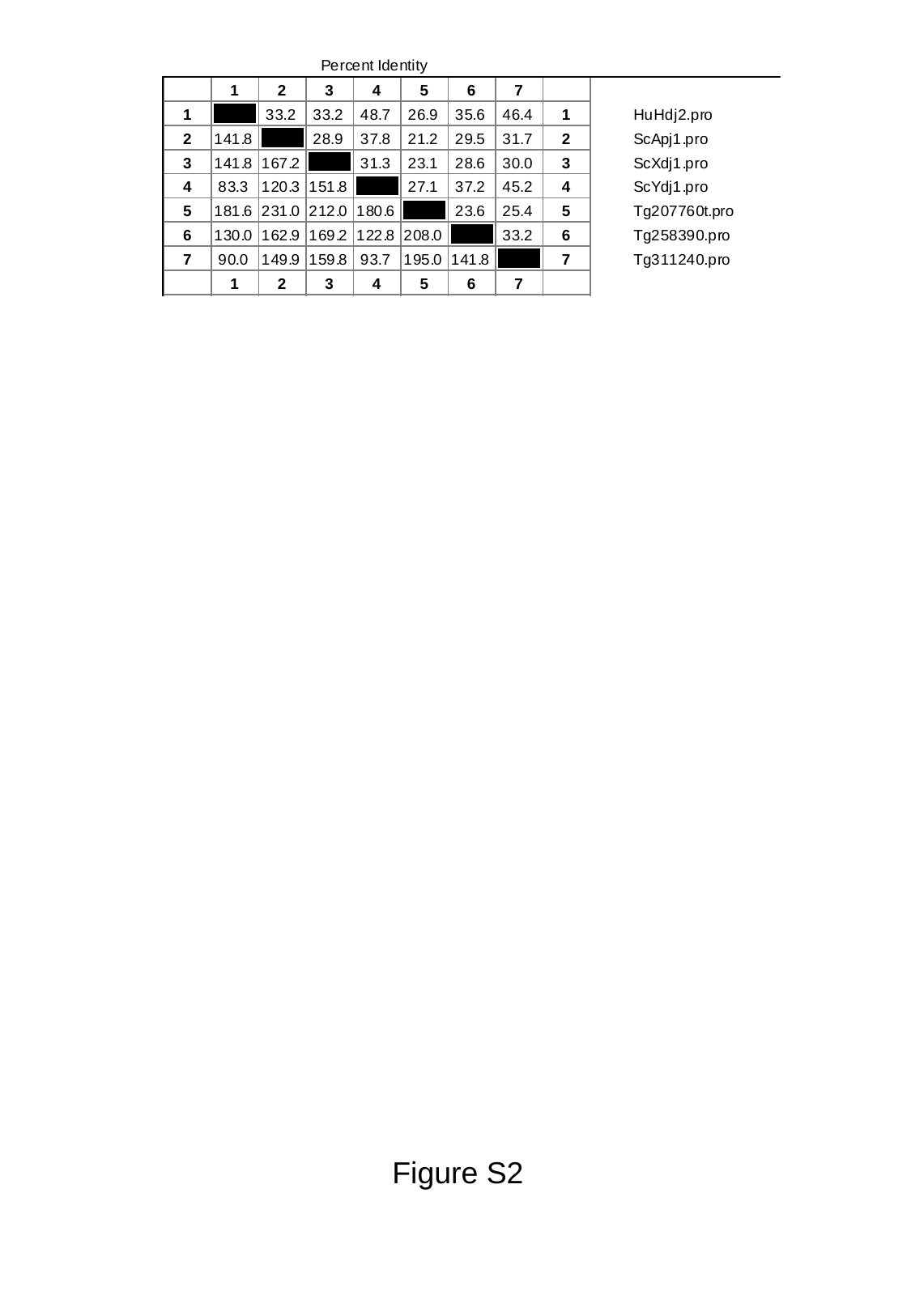

Figure S2

Supplement: Supplementary file 1 [file proteomes-11-00009-s001.zip › FigSRV2.pptx]
